# Synthesis of Blue Gahnite (ZnAl_2_O_4_:Co, Nd): A Cost-Effective Method for Producing Solar-Reflective Pigments for Cool Coatings

**DOI:** 10.3390/ma16041696

**Published:** 2023-02-17

**Authors:** Julia de Oliveira Primo, Dienifer F. L. Horsth, Nayara Balaba, Polona Umek, Fauze J. Anaissi, Carla Bittencourt

**Affiliations:** 1 Chemistry Department, Universidade Estadual do Centro-Oeste, Guarapuava 85040-200, Brazil; 2 Chimie des Interactions Plasma-Surface (ChIPS), Research Institute for Materials Science and Engineering, University of Mons, 7000 Mons, Belgium; 3 Solid State Physics Department, Jožef Stefan Institute, 1000 Ljubljana, Slovenia

**Keywords:** recycled metallic aluminum, near-infrared reflection, circular economy, neodymium, cobalt

## Abstract

Developing strategies for the green synthesis of novel materials, such as pigments for protection from solar radiation, is a fundamental research subject in material science to mitigate the heat island effect. Within this perspective, the current study reports on the synthesis of blue pigments of ZnAl_2_O_4_:M (M = Co^2+^ and Co^2+^/Nd^3+^) using recycled metallic aluminum (discarded can seal) with reflective properties of Near-infrared radiation. The pigments were characterized by XRD, SEM, XPS, UV-Vis, NIR diffuse reflectance spectroscopy, and CIE-1976 L*a*b* color measurements. The wettability of the coatings containing the synthesized pigments was also evaluated. The structural characterization showed that the pigments present the Gahnite crystalline phase. Colorimetric measurements obtained by the CIEL*a*b* method show values correlated to blue pigments attributed to Co^2+^ ions in tetrahedral sites. The pigments exhibit high near-infrared solar reflectance (with R% ≥ 60%), with an enhancement of nearly 20% for the pigment-containing neodymium when applied in white paint, indicating that the prepared compounds have the potential to be energy-saving color pigments for coatings.

## 1. Introduction

Current urban development is a global issue as it triggers the formation of heat islands and increases CO_2_ pollution [1]. It has been reported that urban areas experience higher temperatures due to the Urban Heat Island effect than rural areas [2,3]. Furthermore, as the planet warms, electricity consumption has increased due to spending on cooling equipment. A report on “Space cooling” published by the International Energy Agency estimated that the number of air conditioners worldwide has doubled since 2000, reaching over 2.2 billion units in 2021 [4]. In 2021, total cooling energy demand rose by more than 6.5% globally, with growth in Asia Pacific and Europe hovering around 8–9% [4]. As temperatures rise, it is essential that buildings’ energy codes and local planning include cooling-oriented design strategies, including passive and nature-based solutions, which may reduce cooling needs and prevent heat islands in expanding urban areas. Cool coatings have become superior passive cooling technologies for urban surfaces and areas [2,5,6], developed with materials with high solar reflection in the NIR region [2,7,8,9], which can reduce the energy consumption required for building cooling and improves the thermal comfort of urban living [5].

Inorganic pigments are widely used as cooling materials for residential coatings, which can be developed with high near-infrared reflectance properties for application in cool coatings [10,11]. Since the reflectivity and absorption of pigments are independent, cool pigments may have any color [12]. The recent literature reports focus on developing innovative NIR blue pigments [6,13,14,15,16], becoming a common concern for advancing novel eco-friendly and high NIR reflective blue pigments. The Co^2+^ ion remains the traditional source of blue color in ceramic pigments [17], whereas CoAl_2_O_4_, the cobalt (II) aluminate spinel, is a conventional blue pigment due to its intensely bright blue color. However, producing CoAl_2_O_4_, which contains 33% Co^2+^ by mass, is costly and environmentally damaging [18].

Therefore, we aim to synthesize blue pigments by inserting a low amount of Co (II) cations within a gahnite (ZnAl_2_O_4_) host lattice. The synthesis route for the blue pigment was focused on improving the new pigment’s NIR reflectance value and blue hue by inserting rare earth, the Nd ions, in the aluminate matrix. Rare earth-based NIR reflective pigments have been proposed as viable alternatives to traditional toxic pigments due to their low toxicity [19]. Thus, reducing the amount of Co, the developed pigment allows for both cost-savings and environmental protection.

Herein, we report the synthesis and the influence of rare earth doping on the reflective properties of the pigments based on the gahnite phase. First, acid digestion of can seals was used to recycle aluminum, then as a pigment precursor. Second, the pigments were obtained by the coprecipitation method, followed by calcination at 1000 °C, using starch as fuel. The calcination reaction from recycled aluminum, water, starch, metal salts of zinc, and cobalt was performed in the absence and presence of Nd. The phase structure, morphology, color, and optical properties of powder pigments ZnAl_2_O_4_:M (M = Co^2+^ and Co^2+^/Nd^3+^) were systematically analyzed. Finally, the applicability of these pigments in waterborne acrylic paint (commercial paint) was performed, and the coating’s NIR-reflective, wettability, and color properties were also investigated.

## 2. Materials and Methods

### 2.1. Materials

For the synthesis of the new pigments of ZnAl_2_O_4_:M (M = Co^2+^ and Co^2+^/Nd^3+^), aluminum was recycled from discarded can seal, ZnCl_2_•6H_2_O (Zinc chloride hexahydrate, Dinâmica, reagent grade, 97%), CoCl_2_•6H_2_O (Cobalt (II) chloride hexahydrate, Dinâmica, Gorizia, Italy, reagent grade, 98%), Nd(NO_3_)_3_•6H_2_O (Neodymium (III) nitrate hexahydrate, Sigma Aldrich, St. Louis, MI, USA, reagent grade, 99.9%) were used as precursors. Deionized water was used as a solvent, HCl (Hydrochloric acid, NEON (São Paulo, Brazil), reagent grade, 37.8%) was used for acid digestion of aluminum, NaOH (Sodium hydroxide, NEON, reagent grade 99%) was used to correct the pH, and starch was used as fuel.

### 2.2. Acid Digestion of Metallic Aluminum

The aluminum recycling was performed through the acid digestion of can seals. To obtain each pigment, 1 g of can seal was used, which was washed in water to remove dirt accumulation, then 110 mL of HCl solution (1 mol L^−1^) was added and left reacting at room temperature for 24 h, this being the time required to stabilize the reaction medium.

### 2.3. Synthesis of Gahnite Pigments

**Figure 1:**
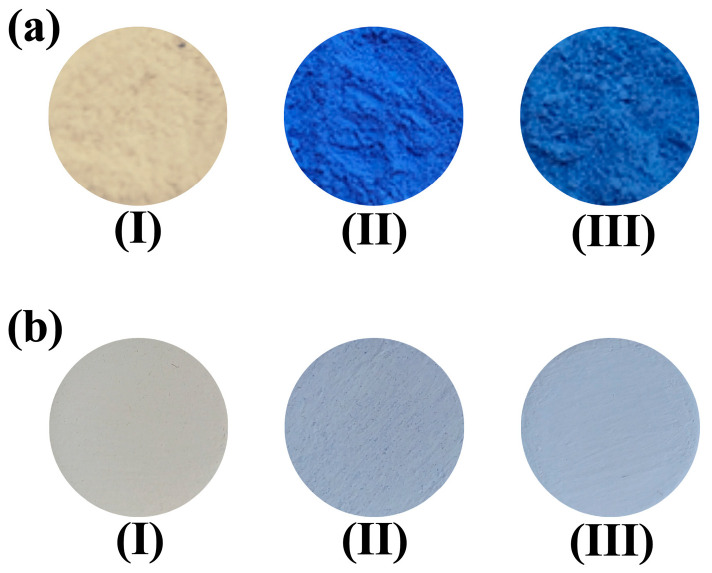
Photographs of the ZnAl_2_O_4_:M (M = Co^2+^ and Co^2+^/Nd^3+^) synthesized pigments: (**a**) in powder form and (**b**) dispersed in commercial white paint. The respective pigments were denoted: as (I) ZnAl_2_O_4_; (II) Co-Gahnite; and (III) CoNd-Gahnite.

The Gahnite samples were prepared by the coprecipitation method, starting from the matrix solution containing the Al^3+^ ions obtained (described in Section 2.2). In each solution, 12.7% (mol%) of zinc (ZnCl_2_) was added to Solution A; therefore, 6.7% of cobalt chloride (mol%) and 0.04% neodymium nitrate (mol%) were added related to aluminum mass into Solution A. The pH of the solutions was corrected to 8 to obtain the boehmite phase (γ-AlOOH) of aluminum oxide-hydroxide, due to its lamellar property, by dripping with NaOH (2 mol L^−1^), then 10 g of starch was added to de solutions and stirred at RT for 10 min. The suspensions were calcined at 1000 °C for 1 h in a 20 °C/ 5 min ramp and pulverized. After calcination, the samples were washed with hot water to remove NaCl. The pigments studied are ZnAl_2_O_4_ and ZnAl_2_O_4_:M (M = Co^2+^ and Co^2+^/Nd^3+^) (Figure 1a).

### 2.4. Preparation of Pigmented Coatings

The obtained gahnite pigments were dispersed into a white waterborne acrylic paint with a solids content by weight of 50.5–52.5% and pH of 8–9 (Paracem^®^ deco matt, prod. Martin Mathys N. V., Zelem, Belgium). The mass % composition of the prepared paint was: 50.0 wt.% commercial white paint, 10.0 wt.% synthesized pigment, and 40.0 wt.% water. The components were agitated for 20 min in mechanical stirring to ensure the complete dispersion of the pigments in the white paint. The paints were coated on 25 × 25 mm polycarbonate surfaces using a brush and dried at room temperature for over 24 h to obtain the coatings (Figure 1b).

### 2.5. Characterization

The crystalline structure of the pigments was analyzed by recording the X-ray powder diffraction (XRD) performed on a Bruker model D2 Phaser (Bruker, Karlsruhe, Germany) with Cu Kα radiation (λ = 1.5418 Å). The morphology of the pigments was examined with a High-Resolution scanning electron microscope HR-SEM Hitachi SU8020 (Hitachi, Tokyo, Japan). The agglomerated particle size distribution histograms of the pigments were performed using Image J (Version 1.53K) [20]. For the SEM analysis, the particulate samples were deposited in carbon tape water. The oxidation state and chemical composition of pigments were determined by X-ray photoelectron spectroscopy (XPS) (Versaprobe PHI 5000, from Physical Electronics, Chanhassen, MN, USA) equipped with a monochromatic Al Kα X-ray source. The spectra were analyzed by CASA-XPS software (Version 2.3.17PR1.1); the binding energies were calibrated using the C1s peak (284.6 eV) of carbon impurities as a reference. Diffuse absorbance spectra after Kubelka–Munk transformation were recorded with a step of 1 nm on a Perkin Elmer Lambda 950 UV-Vis NIR spectrophotometer using an integration sphere at room temperature. Barium sulfate was used as a reference. The colorimetric analyses of the pigment powders before and after they were applied to the PC substrate were measured using a portable colorimeter (3nh, model NR60CP, Shenzhen, China) with a D65 light source. In colorimetric (CIEL*a*b*) analyses, the L* parameter is the brightness, ranging from 0 to 100. The parameter a* between red and green, where +a is prone to red and −a* is prone to green. Meanwhile, the parameter b* indicates the variation between blue and yellow; the +b* value denotes yellow, and the −b* value tends to be blue [21]. Both a* and b* range from −128 to +128. The contact angle (CA) of the surfaces painted with the synthesized pigments was measured by a contact angle meter (Attension Theta, Biolin Scientific, Gothenburg, Sweden) using a water droplet with a volume of ~5 µL under ambient conditions (25 °C). The CA values are the averages of ten measuring points on each surface.

The optical reflectance of the pigment powders and corresponding coatings was measured using a UV-Vis-NIR spectrophotometer (Perkin Elmer Lambda 950, Waltham, MA, USA). As a baseline standard, BaSO_4_ was used to measure the optical properties of the samples between 300 and 2500 nm. The NIR solar reflectance (R*) of the pigments and coating in the wavelength range of 750–2500 nm was obtained by the following Equation (1):

(1) $$R^{*}=\frac{\int_{750}^{2500}r\left(\lambda \right)i\left(\lambda \right)d\lambda }{\int_{750}^{2500}i\left(\lambda \right)d\left(\lambda \right)}$$

where *r*(*λ*) is the spectral reflectance obtained from the experimental, and *i*(*λ*) is the spectral irradiance obtained from the standard of ASTM G173-03 reference spectra (W∙m^−2^∙nm^−1^) [2,6,9].

## 3. Results and Discussion

### 3.1. Characterization of the Powder Pigments

**Figure 2:**
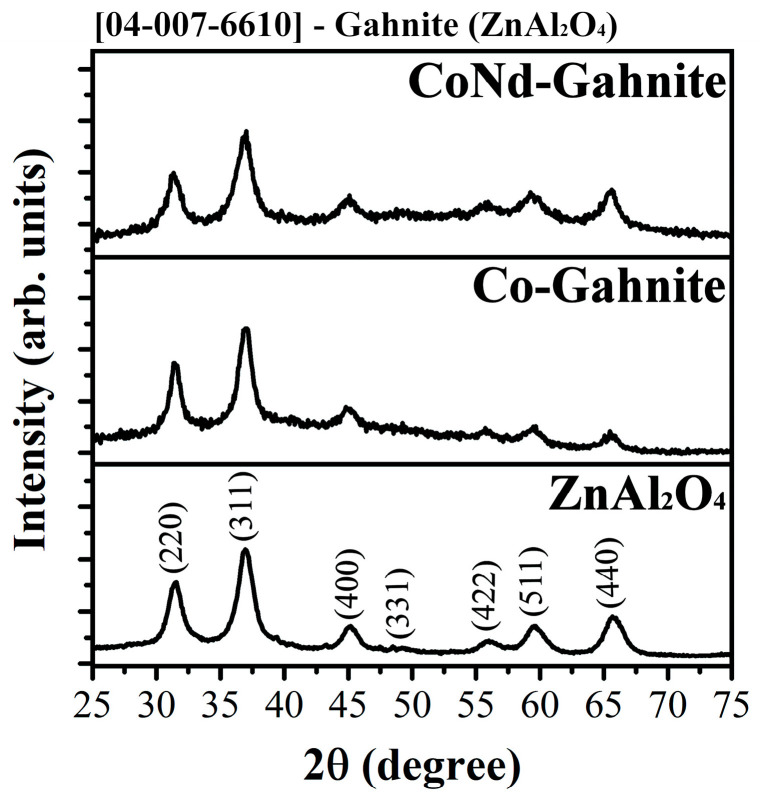
X-ray powder diffraction patterns of ZnAl_2_O_4_ (M = Co^2+^ and Co^2+^/Nd^3+^) pigments (showing the Gahnite structure of ZnAl_2_O_4_).

Figure 2 shows the X-ray diffraction patterns (XRD) of the three pigments synthesized ZnAl_2_O_4_, ZnAl_2_O_4_:Co, and ZnAl_2_O_4_:Co; Nd obtained after annealing in air at 1000 °C for 1 h. The synthesized pigments consist mainly of gahnite, ZnAl_2_O_4_ (ICDD card number 96-900-7024), with two prominent peaks appearing at 31.2° and 36.8°, which are associated with (220) and (311) the crystallographic planes. These are characteristics of a cubic crystal structure belonging to the spinel compounds with the general formula AB_2_O_4_, where A and B are predominantly zinc (Zn) and aluminum (Al), bi- and trivalent, respectively [22,23]. The diffraction peaks confirm that the calcination temperature of 1000 °C is suitable for obtaining monophasic gahnite pigments, which the substitution occurred in the tetrahedral sites occupied by Zn^2+^ ions; this process is favored by the similar ionic radii of IV-fold coordinated Co^2+^ and Zn^2+^, 0.58 Å and 0.60 Å, respectively [24]. Furthermore, XRD results showed that the amount of Nd ions inserted into the host lattice did not affect the gahnite phase. The XRD results indicated that 1000 °C is an optimum synthesis temperature to obtain a monophasic structure of ZnAl_2_O_4_ by the synthesis route proposed in this work.

**Figure 3:**
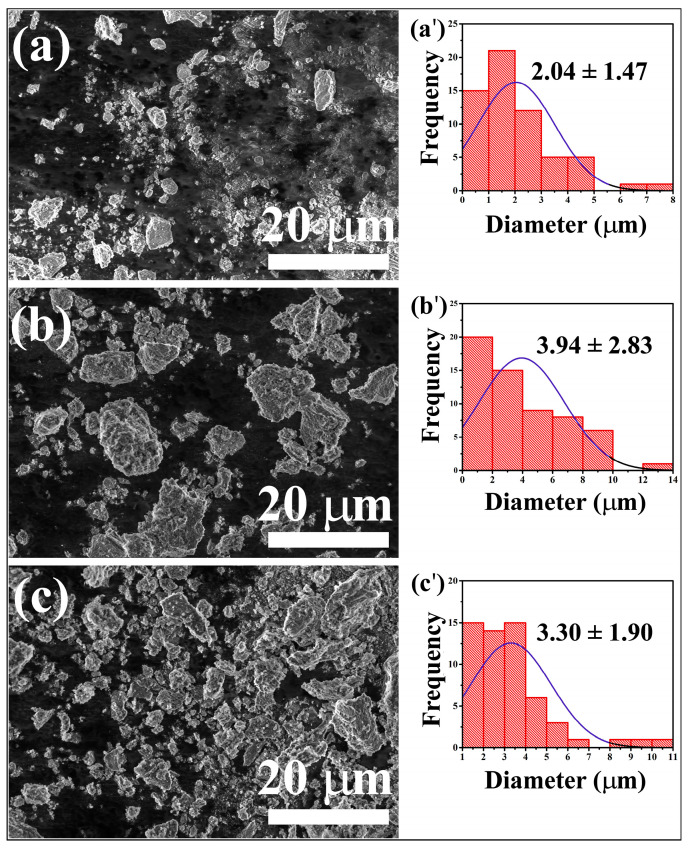
SEM images of ZnAl_2_O_4_:M (M = Co^2+^ and Co^2+^/Nd^3+^) pigments: (**a**) ZnAl_2_O_4_, (**b**) Co-Gahnite, and (**c**) CoNd-Gahnite. The corresponding images (**a′**), (**b**′), and (**c**′) are related to the particle size aglomerates distribution histograms of the pigments.

The microstructure analysis of the samples was carried out using high-resolution scanning electron microscopy (SEM) to determine the grain size and surface morphology. In Figure 3, SEM images display the pigments with a rough surface that tends to agglomerate into small irregular shapes after annealing at 1000 °C for 1 h, suggesting a microcrystalline nature of the pigments. The agglomerated particles are not uniform, ranging in size from 0.6–8 μm, 0.8 to 14 μm, and 1–11 μm for ZnAl_2_O_4_, Co-Gahnite, and CoNd-Gahnite, respectively.

**Figure 4:**
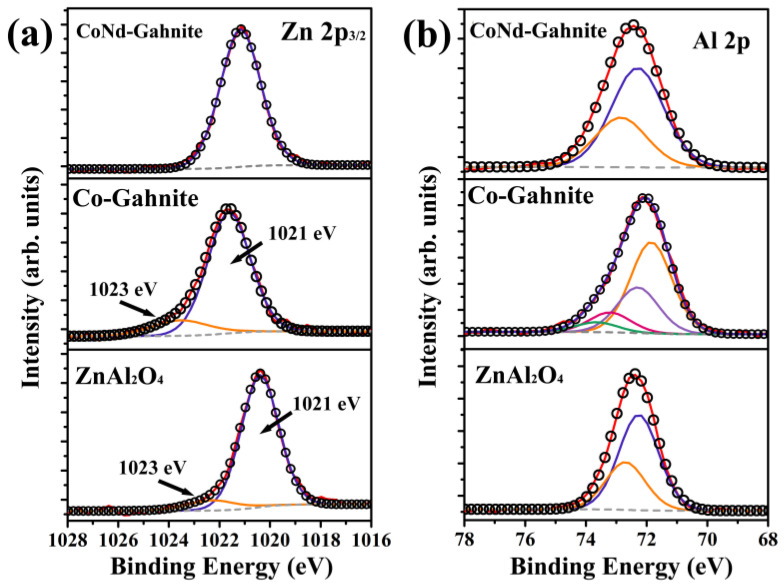
XPS analysis of ZnAl_2_O_4_, Co-Gahnite, and CoNd-Gahnite pigments. The experimental solid line and fitted curves of high-resolution XPS spectra of (**a**)Zn 2p_1/2_; and (**b**) Al 2p.

To study the electronic structure of the major elements, near-surface region, the high energy resolution photoelectron spectra of Zn 2p and Al 2p core levels are shown in Figure 4; the spectra were calibrated based on the reference peak of C 1s at 284.6 eV from carbon contamination. The Zn 2p_3/2_ XPS spectra were deconvoluted into two components, appearing in the range 1020–1021 eV and 1022–1023, assigned to the tetrahedrally (Td) and octahedrally (Oh) coordinated zinc [23,25], indicating that the Zn^2+^ ions occupy the site in an inverse spinel configuration in the ZnAl_2_O_4_ and Co-Gahnite pigments obtained. The ratio of the areas under these components suggests a higher percentage of inversion in Zn^2+^ site occupancy for the Co-Gahnite sample, whereas, for the pigment CoNd-Gahnite the Zn^2+^ ions are located in tetrahedral positions only (Figure 4a), denoting that the insertion of Nd ions into Gahnite structure reduces the distortion in the crystalline structure towards an inverse spinel. Similar results were observed by [23]. The XPS Al 2p core level spectra were fitted by the two components of the 2p doublet for the ZnAl_2_O_4_ and CoNd-Gahnite samples, indicating that the Al^3+^ ions occupy the inverted tetrahedral coordination site (Figure 4b) while for the Co-Gahnite two doublets used to fit the Al 2p peak indicating that Al^3+^ ions occupy more than one coordination site, i.e., the octahedral and the tetrahedral coordination site.

The high energy resolution photoelectron spectra of O 1s are shown in Figure S1. The O 1 s peak is fitted with two components (Figure S1). The component centered at 530.8 eV was assigned to oxygen atoms participating in Zn-O and Al-O bonds in the ZnAl_2_O_4_ lattice. The low-intensity component centered at 532.7 eV indicates an oxygen deficiency in the ZnAl_2_O_4_ crystal lattice [26]. The fitting analysis of the Co 2p core level is shown in Figure S2, which was performed to evaluate the oxidation state of cobalt in the Gahnite structure. The XPS analysis indicated that the most prominent cobalt species present in the Gahnite structure was Co_3_O_4_ [27], which confirmed the presence of cobalt in mixed oxidation states of 2+ and 3+ at the surface of the agglomerates.

**Figure 5:**
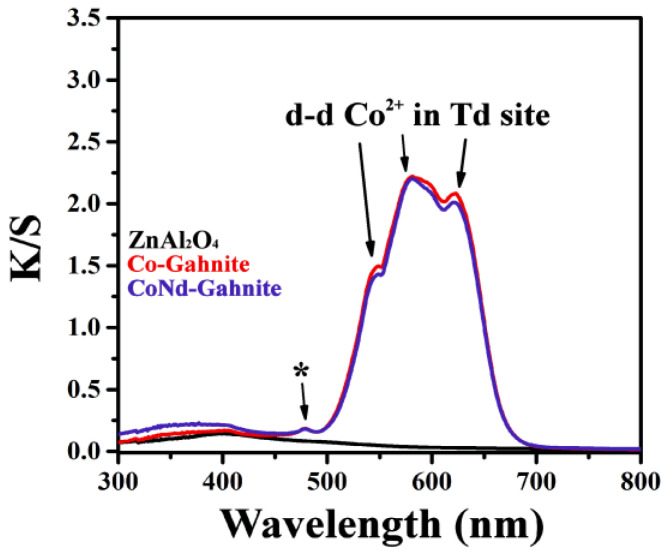
K/S absorption spectra of ZnAl_2_O_4_:M (M = Co^2+^ and Co^2+^/Nd^3+^) pigments, calcined at 1000 °C.

The K/S absorption spectra were recorded in the wavelength range of 300–800 nm to study the optical properties of the pigments (Figure 5). The graphic shows the triplet d-d band located at 548 (green region), 582 (yellow-orange region), and 622 nm (red region), which gives rise to the blue coloration [28]; this triplet is assigned to the ^4^A_2_(4F) → ^4^T_1_(4P) transition of tetrahedrally coordinated Co^2+^ ions [17,25,29,30], attributed to a Jahn-Teller distortion of the tetrahedral structure [28]. Thus, the pigments’ blue color is primarily due to this strong d–d transition of tetrahedral Co^2+^ between 500 and 675 nm. The low-intensity peaks around 478 nm (indicated in the image by the symbol *) are related to the spin-forbidden transition, which was attributed to transitions between octahedral and tetrahedral sites [13]; this peak was identified for similar material, Co-doped ZnAl_2_O_4_ obtained at 1000 °C [25,29]. The pigment-containing neodymium showed no changes in this spectrum region, with no significant decrease in the intensity of the absorption band around 500–650 nm.

Table 1 shows the measured CIEL*a*b* parameter for the pigments in powder form calcined at 1000 °C. From the colorimetric point of view, both pigments containing cobalt are in the red/blue quadrant (+a/−b), and with the insertion of Nd ions, the blue hue enhanced, i.e., the b* coordinate value increased. The brightness (L*) of the ZnAl_2_O_4_ decreased with the cobalt insertion, and the pigment Nd-bearing is the lighter blue pigment compared to the pigment bearing only with Co. The b* chromatic parameter also shifts versus the composition of the pigments, in which the pigment CoNd-Gahnite showed a higher b* contrast; in other words, the pigment with a higher blueish hue (b* from −34.39 to −36.89). It has been shown in these studies that a small amount of rare earth can alter the hue of the pigments, which can be explored further for obtaining new colors.

### 3.2. NIR Reflectance Properties of the Powder Pigments

**Figure 6:**
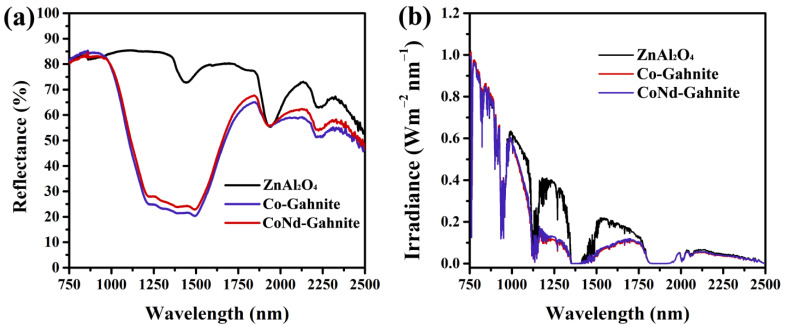
(**a**) Diffuse reflectance spectra of the synthesized pigments in powder form, and (**b**) NIR solar reflectance spectrum of the powder pigments adjusted to the standard solar spectrum.

To evaluate the effectiveness of the materials as cool pigments and, consequently, alleviating the urban heat island effect, the NIR reflectance of the blue powder pigments ZnAl_2_O_4_:M (M = Co^2+^ and Co^2+^/Nd^3+^) was measured at 750–2500 nm (Figure 6). Additionally, ZnAl2O4 has been analyzed to determine the NIR reflectance associated with cobalt pigments (blue pigments). As shown in Figure 6a, it can be observed that the insertion of the Co ions decreases the solar reflectance compared to the ZnAl_2_O_4_ reflectance spectra; this is due to significant absorption in the 1200–1600 nm of d-d transitions for tetrahedral Co^2+^ [31], giving a significant absorption in the NIR region. The NIR solar reflectance curves of the powder pigment samples were calculated by ASTM standard G173-03 (Figure 6b). The average solar reflectance of the CoNd-Gahnite pigment had a slight improvement in the NIR reflectance compared to Co-Gahnite (Table 1); these results may be due to the low Nd amount present in the matrix. However, both pigments’ average NIR solar reflectance did not fall below 60%. This shows its ability to serve as a cool pigment with improved thermal insulation performance in practical applications. A comparison of synthetic pigment samples with other blue compounds is presented in Table 2. The results show that pigments containing rare earth are potential candidates for cool pigments, which alter the pigments’ hue and improve the final pigment’s NIR solar reflectance properties.

Several factors can affect the reflectivity of materials, including particle size, morphology, and uniformity of particle size distribution [1,32]. According to the Kubelka–Munk theory, particles’ amount of infrared radiation scattered increases with decreasing particle size [1]. It can be seen from Figure 3 that the particle size of the CoNd-Gahnite sample decreases compared to Co-Gahnite, which is favorable for light scattering.

**Table 2:** Color Coordinates and NIR Reflectivity of Co-Gahnite, CoNd-Gahnite, and other blue pigments with the synthesis method employed.

Sample	Synthetic Method	Color Coordinates			Color from Color Coordinates	NIR Reflectance (R%)	Reference
		L*	a*	b*			
CoAl_2_O_4_	Commercial product	44.8	2.1	−32.7	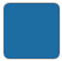	29.0	[6]
YIn_0.9−x_Mn_0.1_Zn_0.4_O_3−δ_	Solid-state	46.58	3.01	−42.49	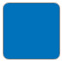	72.08	[6]
Sr_0.6_Nd_0.4_CuSi_4_O_10+δ_	Sol-gel	68.7	−6.6	−24	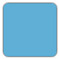	68.54	[16]
Yin_0.9_Mn_0.1_O_3_-ZnO	Sol-gel	49.94	−0.88	−40.55	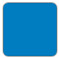	70.0	[33]
CaAl_11.7_Co_0.1_Ti_0.2_O_19_	Solid-state	64.89	3.88	−40.30	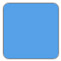	69.22	[15]
Zn_0.9_Co_0.1_Al_2_O_4_	Combustion synthesis	67.9	−3.7	−39.0	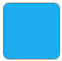	63.0	[34]
Co-Gahnite	Coprecipitation followed by solution combustion synthesis	43.83	11.86	−34.39	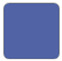	62.04	This work
CoNd-Gahnite	Coprecipitation followed by solution combustion synthesis	45.36	8.12	−36.89	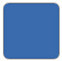	63.01	This work

### 3.3. Coating Studies

The color properties of the pigments applied in a commercial white paint were investigated further by colorimetry. As shown in Table 3, white paint played a critical role in the color properties of the pigments. Table 3 shows that the brightness (L*) increased significantly with the pigments dispersed in the paint, as long as decrease the chromatic parameter (a* and b*), due to the white matrix of the commercial paint being TiO_2_, which tends to lighten the blue hue of the dispersed pigments.

**Figure 7:**
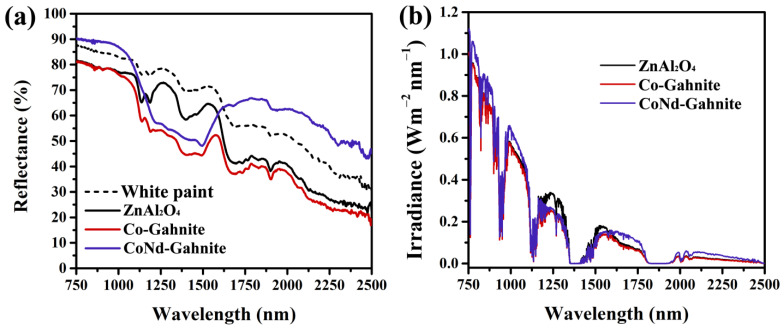
(**a**) Diffuse reflectance spectra of the synthesized pigments dispersed in white commercial paint; and (**b**) NIR solar reflectance spectrum of the coatings adjusted to the standard solar spectrum.

The Near Infrared spectra were also obtained for the pigments applied in waterborne acrylic paint to evaluate the performance of the pigments in a coating (Figure 7). The commercial paint used in this study contains the white pigment titanium dioxide, as observed in our previous work [35], which can influence the NIR reflectance of the surface coatings due to its high solar reflectivity [5]. The NIR solar reflectance curves of the coatings were calculated by ASTM standard G173-03 (Figure 7b). As indicated in Table 3, there is a significant increase in R% for the pigment-containing Nd dispersed in paint, which produced a blue-colored pigment with almost 20% enhancement in NIR reflectance; this may be due to the CoNd-Gahnite pigment having a better dispersion of the particles compared to Co-Gahnite in acrylic-based paint, thus reducing the degree of agglomeration. The results indicate that synthetic pigments containing rare earth elements are promising for applying cool coatings.

**Figure 8:**
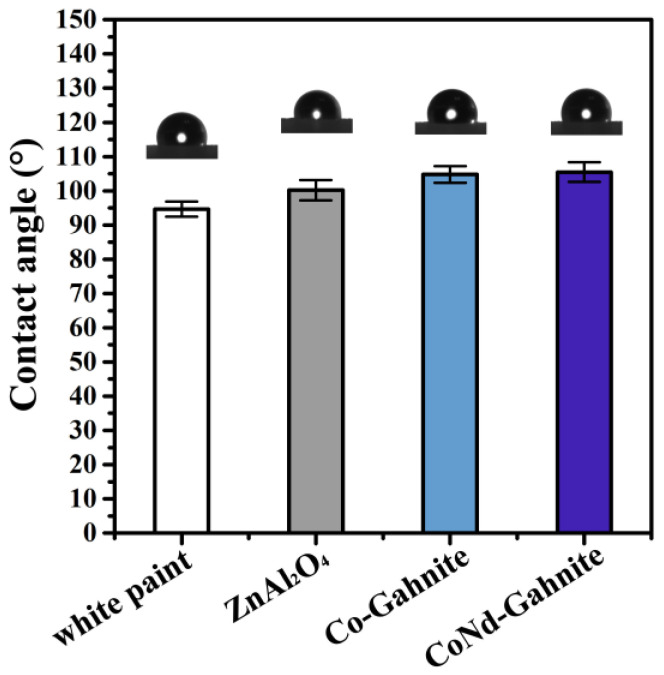
Histogram showing the contact angle values of the pigment in coatings applied on a polycarbonate substrate.

Self-cleaning infrared-reflective surfaces are required to prevent dust from adhering to the film coating and reducing infrared reflectivity [36,37,38]. A solid surface’s coating properties can be measured simply by dropping water on the surface. Figure 8 shows the CA of the pigments coated in white commercial paint. Water wettability measurements results showed that the CAs of the coatings containing the synthesized pigments are all larger than 100°; the ZnAl_2_O_4_ coating surface had a CA of 100.2° ± 3.0°, while the commercial white paint showed a CA of 94.7° ± 2.2°. The surfaces containing the blue pigments increased the CA value, indicating a CA of 104.8° ± 2.4° and 105.5° ± 2.9° for Co-Gahnite and CoNd-Gahnite, respectively, showing that the insertion of rare earth in the composition can also improve the hydrophobic properties of the infrared-reflective surface. These preliminary results show that the coatings developed in this study, containing the synthesized blue pigments, exhibit a hydrophobic property. This enables them to keep the surface clean and maintain its reflective properties.

## 4. Conclusions

Blue pigments ZnAl_2_O_4_:M (M = Co^2+^ and Co^2+^/Nd^3+^) were successfully obtained using recycled aluminum from seals as a precursor to obtaining the Gahnite crystalline phase. In this work, starch was used as a fuel during the calcination step; this natural additive makes the synthesis more environmentally friendly. In conjunction with recyclable aluminum, the use of starch for preparing materials to save energy and the environment matches the circular economy concept. The pigments presented high crystallinity, homogeneity, and single crystalline phase from calcination at 1000 °C. They present the Gahnite-type structure, even with the insertion of Co and Nd ions into the ZnAl_2_O_4_ matrix. The synthesized pigments had good dispersion with acrylic-based paint, coloring the white paint and enhancing the Near reflectance properties. Therefore, the blue synthesized pigments are sustainable candidates to substitute the expensive commercial cobalt blue pigments and have the potential to be used as cool coatings for energy saving.

## Figures and Tables

**Table 1:** Color coordinates, NIR reflectance of the powdered pigments.

Sample	Color Coordinates			Color from Color Coordinates	NIR Reflectance (R%)
	L*	a*	b*		
ZnAl_2_O_4_	79.63	5.15	12.45	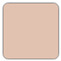	81.75
Co-Gahnite	43.83	11.86	−34.39	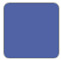	62.04
CoNd-Gahnite	45.36	8.12	−36.89	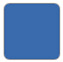	63.01

**Table 3:** Color coordinates, NIR reflectance of the pigments dispersed in commercial white paint.

Sample	Color Coordinates			Color from Color Coordinates	NIR Reflectance (R%)
	L*	a*	b*		
ZnAl_2_O_4_	89.69	1.13	9.22	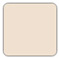	69.85
Co-Gahnite	67.13	2.00	−27.09	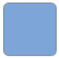	64.97
CoNd-Gahnite	68.72	−0.04	−28.77	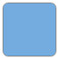	76.02

## Data Availability

Not applicable.

## Supplementary Materials

The following supporting information can be downloaded at: https://www.mdpi.com/article/10.3390/ma16041696/s1. XPS analysis of ZnAl_2_O_4_, Co-Gahnite, and CoNd-Gahnite pigments.
