# Supplementary material for: Synthesis of Blue Gahnite (ZnAl2O4:Co, Nd): A Cost-Effective Method for Producing Solar-Reflective Pigments for Cool Coatings
Source: Materials (Basel). 2023 Feb 17;16(4):1696. doi: 10.3390/ma16041696 (PMC9963338; doi:10.3390/ma16041696)
Supplement: Supplementary file 1 [file materials-16-01696-s001.zip › materials-2161444-supplementary.pdf]

Supplementary Material

Synthesis of Blue Gahnite ($ZnAl_2O_4$:Co, Nd): A cost-effective method for producing solar-reflective pigments for cool coatings

Julia de Oliveira Primo [1], Dienifer F. L. Horsth [1,2], Nayara Balaba [1], Polona Umek[3], Fauze Jacó Anaissi [1] and Carla Bittencourt [2,*]

[1] Chemistry Department, Universidade Estadual do Centro-Oeste, Guarapuava 85040-200, Brazil

[2] Chimie des Interactions Plasma-Surface (ChIPS), Research Institute for Materials Science and Engineering, University of Mons, 7000 Mons, Belgium

[3] Solid State Physics Department, Jožef Stefan Institute, 1000 Ljubljana, Slovenia

* Correspondence: carla.bittencourt@umons.ac.be

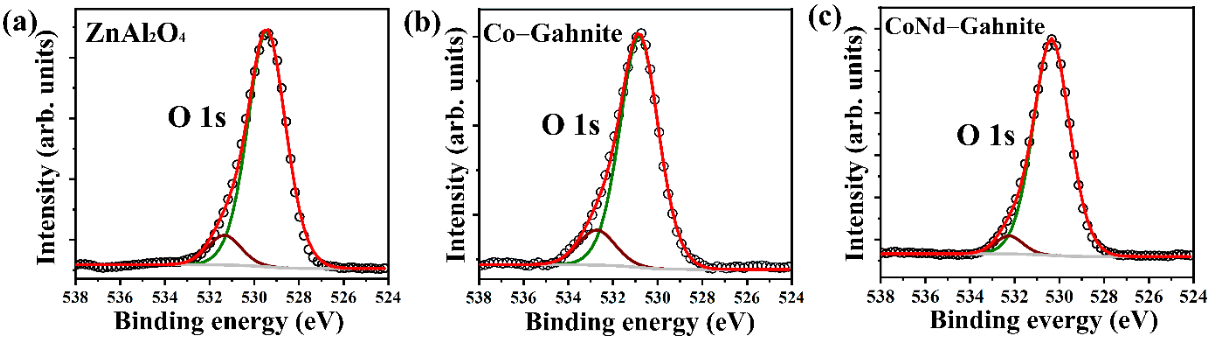

Figure S1. XPS analysis of $ZnAl_2O_4$, Co-Gahnite, and CoNd-Gahnite pigments. The experimental solid line and fitted curves of high-resolution XPS spectra of O 1s. (a) $ZnAl_2O_4$; (b) Co-Gahnite; (c) CoNd-Gahnite.

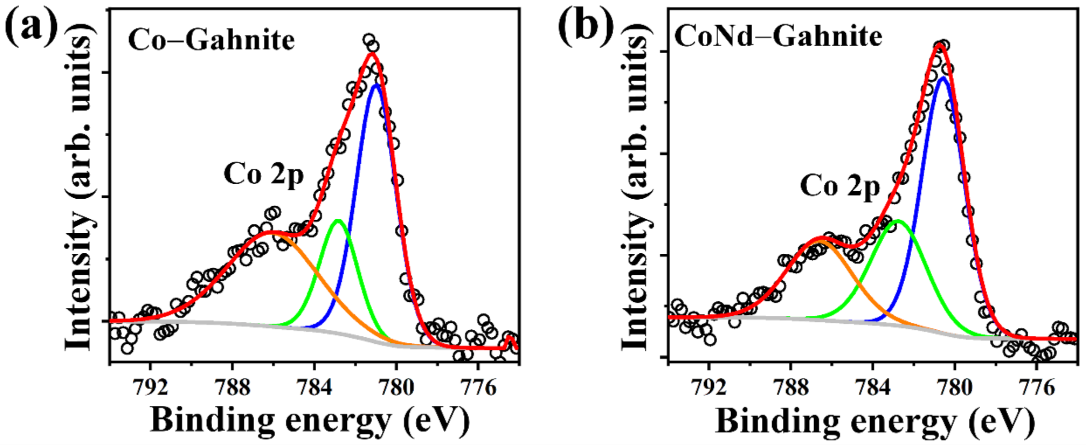

Figure S2. XPS analysis of $ZnAl_2O_4$, Co-Gahnite, and CoNd-Gahnite pigments. The experimental solid line and fitted curves of high-resolution XPS spectra of Co 2p. (a) Co-Gahnite; (b) CoNd-Gahnite.
